# Localizing wild chimpanzees with passive acoustics

**DOI:** 10.1002/ece3.8902

**Published:** 2022-05-07

**Authors:** Anne‐Sophie Crunchant, Jason T. Isaacs, Alex K. Piel

**Affiliations:** ^1^ School of Biological and Environmental Sciences Liverpool John Moores University Liverpool UK; ^2^ Department of Computer Science California State University Channel Islands Camarillo California USA; ^3^ Department of Anthropology University College London London UK

**Keywords:** acoustic array, ALS, apes, conservation method, error

## Abstract

1. Localizing wildlife contributes in multiple ways to species conservation. Data on animal locations can reveal elements of social behavior, habitat use, population dynamics, and be useful in calculating population density. Acoustic localization systems (ALS) are a non‐invasive method widely used in the marine sciences but not well established and rarely employed for terrestrial species.
2. We deployed an acoustic array in a mountainous environment with heterogeneous vegetation, comprised of four custom‐built GPS synchronized acoustic sensors at about 500 m intervals in Issa Valley, western Tanzania, covering an area of nearly 2 km^2^. Our goal was to assess the precision and error of the estimated locations by conducting playback tests, but also by comparing the estimated locations of wild chimpanzee calls with their true locations obtained in parallel during follows of individual chimpanzees. We assessed the factors influencing localization error, such as wind speed and temperature, which fluctuate during the day and are known to affect sound transmission.
3. We localized 282 playback sounds and found that the mean localization error was 27 ± 21.8 m. Localization was less prone to error and more precise during early mornings (6:30 h) compared to other periods. We further localized 22 wild chimpanzee loud calls within 52 m of the location of a researcher closely following the calling individuals.
4. We demonstrate that acoustic localization is a powerful tool for chimpanzee monitoring, with multiple behavioral and conservation applications. Its applicability in studying social dynamics and revealing density estimation among many others, especially but not exclusively for loud calling species, provides an efficient way of monitoring populations and informing conservation plans to mediate species loss.

## Graphical abstract

We deployed an acoustic array comprised of four custom‐built GPS synchronized acoustic sensors in the Issa Valley, Tanzania. We localized 282 playback sounds with a mean localization error of 27 ± 21.8 m and 22 wild chimpanzee loud calls within 52 m of the location of a researcher closely following the calling individuals. Acoustic localization is a powerful tool for species monitoring with multiple behavioral and conservation applications.

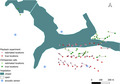

## 1 INTRODUCTION

Localizing animals can help answer questions related to species conservation. The application of this method is highly variable and includes informing on social behavior, habitat use, population dynamics, and estimations of abundance and density (Blumstein et al., 2011; Rhinehart et al., 2020). Direct visual observations of animals are often difficult if they are cryptic, elusive, nocturnal, live in dense vegetation, or range widely. For decades, researchers have relied on animal‐borne loggers to remotely track animals that otherwise elude traditional visual observation methods (Kays et al., 2015; Millspaugh & Marzluff, 2001). However, attaching loggers can be an invasive method and is controversial. It often requires darting and capturing the targeted animal, which can be a stressful event for the animal and can affect subsequent behavior and survival (reviewed in Wilson & McMahon, 2006). Initially restricted to larger animals and offering limited resolution movement data, recent improvements in loggers have resulted in better location resolution and movement accuracy, and from far smaller devices (Kays et al., 2015). Nonetheless, these loggers can be expensive and ultimately are limited by power (battery) capacity, with collars/batteries requiring changing at regular intervals. Alternatively, for those species that have large home ranges and make loud calls, researchers can use acoustic localization to monitor animals, by exploiting sounds that can travel long distances.

Acoustic localization uses the time difference of arrival (TDOA) of sounds to multiple (time synchronized) sensors to estimate the sound origin location, following multilateration (Blumstein et al., 2011; Spiesberger & Fristrup, 1990). Location accuracy varies as a function of inter‐sensor distances. Furthermore, localization can be limited in terms of applicability and more challenging for widely spaced callers, as it would require a larger number of sensors. Sound transmission and thus localization can also be impacted by environmental variables such as high temperature, high wind speed, and vegetation—all of which can distort acoustic signals and affect the signal‐to‐noise ratio (SNR), whereas in noisy environments target signals can overlap with other sounds. Lastly, sensor time synchronization accuracy and recording sample rate can bias the estimation of TDOA and lead to inaccurate localizations (reviewed in Rhinehart et al., 2020). Despite its ubiquity in marine mammalogy, deployment of acoustic localization systems (ALS) is not as common with bird or terrestrial mammal systems. In early studies applying ALS to birds, researchers synchronized acoustic sensors by deploying thousands of meters of cable (Fitzsimmons et al., 2008; Mennill et al., 2006) before later developing wireless time‐synchronized arrays (Collier et al., 2010; Mennill et al., 2012).

Initially pioneered in the marine sciences, early ALSs exploited low attenuation characteristics in underwater sound (Spiesberger & Fristrup, 1990; Stafford et al., 1998). Comparatively, fewer ALS deployments in terrestrial systems have been conducted, likely because of obstacles (i.e., trees) that attenuate sounds and because sound propagates better in water than in the air. Moreover, habituation of animals or else use of other remote sensing devices (e.g., camera traps) also mean alternative means for data collection for ground‐dwelling species. Terrestrial studies have mainly focused on birds (Collier et al., 2010; Mennill et al., 2006, 2012; Wang et al., 2005) and recently on some loud calling mammals, such as orangutans (*Pongo pygmaeus wurmbii*) (Spillmann et al., 2015), elephants (*Loxodonta cyclotis*) (Hedwig et al., 2018; Wrege et al., 2017), and wolves (*Canis lupus*) (Kershenbaum et al., 2019; Papin et al., 2018).

The aim of the current study was to evaluate a custom‐made ALS composed of four GPS time‐synchronized acoustic sensors to localize wild chimpanzees (*Pan troglodytes schweinfurthii*) in western Tanzania. While the system that we describe here is not commercial nor is that an ultimate goal, our successful design, and deployment reflects the feasibility and applicability of off‐the‐shelf systems to broader questions in behavioral biology. There are commercial options available for such work (namely, Song Meters (Wildlife Acoustics, 2022)), but these systems are either expensive or lack the flexibility in design necessary for adapting them to novel applications. Other and more affordable options are being introduced regularly, for example, CARACAL (Wijers et al., 2019), but offer a lower sensitivity (i.e., detecting fewer calls) and thus require more acoustic sensors and consequently a higher quantity of data to analyze (Smith et al., 2021).

Broadly, chimpanzees are wide‐ranging and rely on loud calls that can travel hundreds of meters to coordinate movement (Fedurek et al., 2014; Gruber & Zuberbühler, 2013; Uhlenbroek, 1996). Loud calls range in frequency from ~200 to ~1800 Hz (Marler & Hobbett, 1975) and are produced in different contexts such as movement coordination within and between parties but also during agonistic events. Studies across communities have consistently found bimodal peaks of calling, generally when chimpanzees leave their nest sites in the morning and when arriving at subsequent nesting sites in the evening (e.g., Crunchant et al., 2021; Piel, 2018; Wilson et al., 2007). Furthermore, there is a seasonal difference in calling behavior, with chimpanzees vocalizing more during the late dry and early wet seasons (Crunchant et al., 2020). Call rate is influenced by socio‐ecological factors such as sex, time of day, the presence of a swollen parous female, the proportion of time spent in a vegetation type, and the proportion of time spent traveling. Males vocalize twice as often as females during the late dry season (respectively 1.91 ± 0.12 and. 0.84 ± 0.18 calls per hour) (Crunchant et al., 2021). Our goal was to assess the precision and error of the estimated locations by conducting playback sound experiments, and also by comparing the estimated locations of actual wild chimpanzee calls with the true (ground) locations obtained in parallel with focal follows. We explore the factors influencing the localization error, such as wind speed and temperature that fluctuate during the day, and influence sound attenuation (Harris, 1966). We demonstrate the potential of ALS for localizing wild chimpanzees and discuss the behavioral and conservation applications for this emerging census technique with terrestrial loud calling species.

## 2 METHODS

### 2.1 Study site and study subjects

**FIGURE 1:**
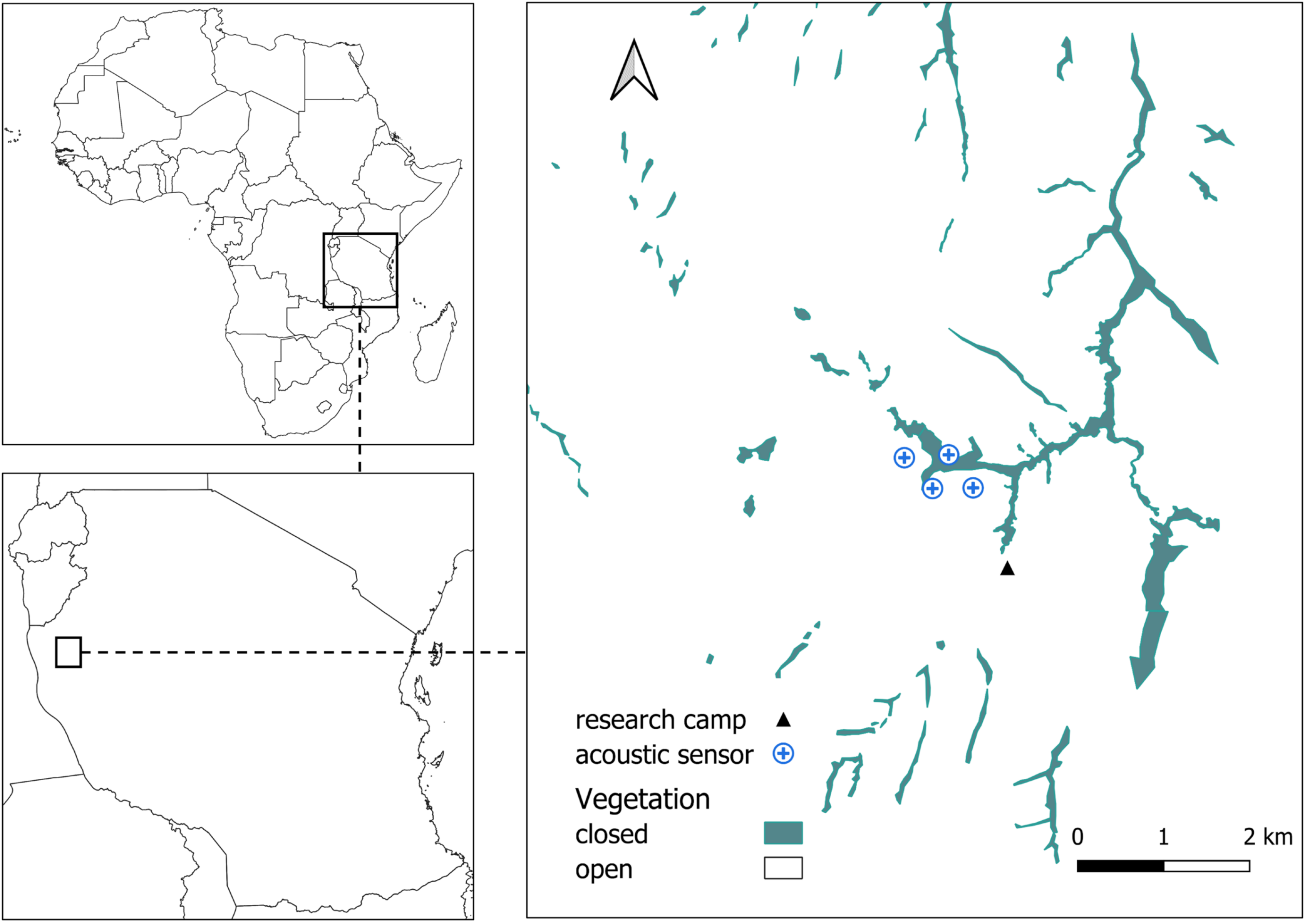
Acoustic localisation system in the Issa Valley, Western Tanzania

We conducted the 3‐month study between August and October 2019, in the Issa Valley, western Tanzania. The study site of about 70 km^2^ is comprised of a series of riverine valleys separated by steep mountains and flat plateaus (Figure 1). Vegetation is dominated by miombo woodland and also includes grassland, swamp, and riparian forest. Despite the mosaic nature of the landscape, chimpanzees spend the majority of their time either in woodland or forest. Therefore for analyses, we collapsed vegetation categories into “open” (woodland, grassland, and swamp) and “closed” (riparian forest). Over the study period, it rained 132.8 mm and temperatures ranged from 14.6 to 32°C.

Chimpanzee habituation in Issa started in 2008 and chimpanzees were fully habituated in September 2018 with nest to nest follows. When the study began, the habituated Issa community comprised nine adult females, seven adult males, four subadult males, one subadult female, three juveniles, and four infants. Two individuals (one female and an infant) were killed, and a female gave birth during the study period. The Issa community has a home range ≥55 km^2^.

### 2.2 Acoustic localization system

**FIGURE 2:**
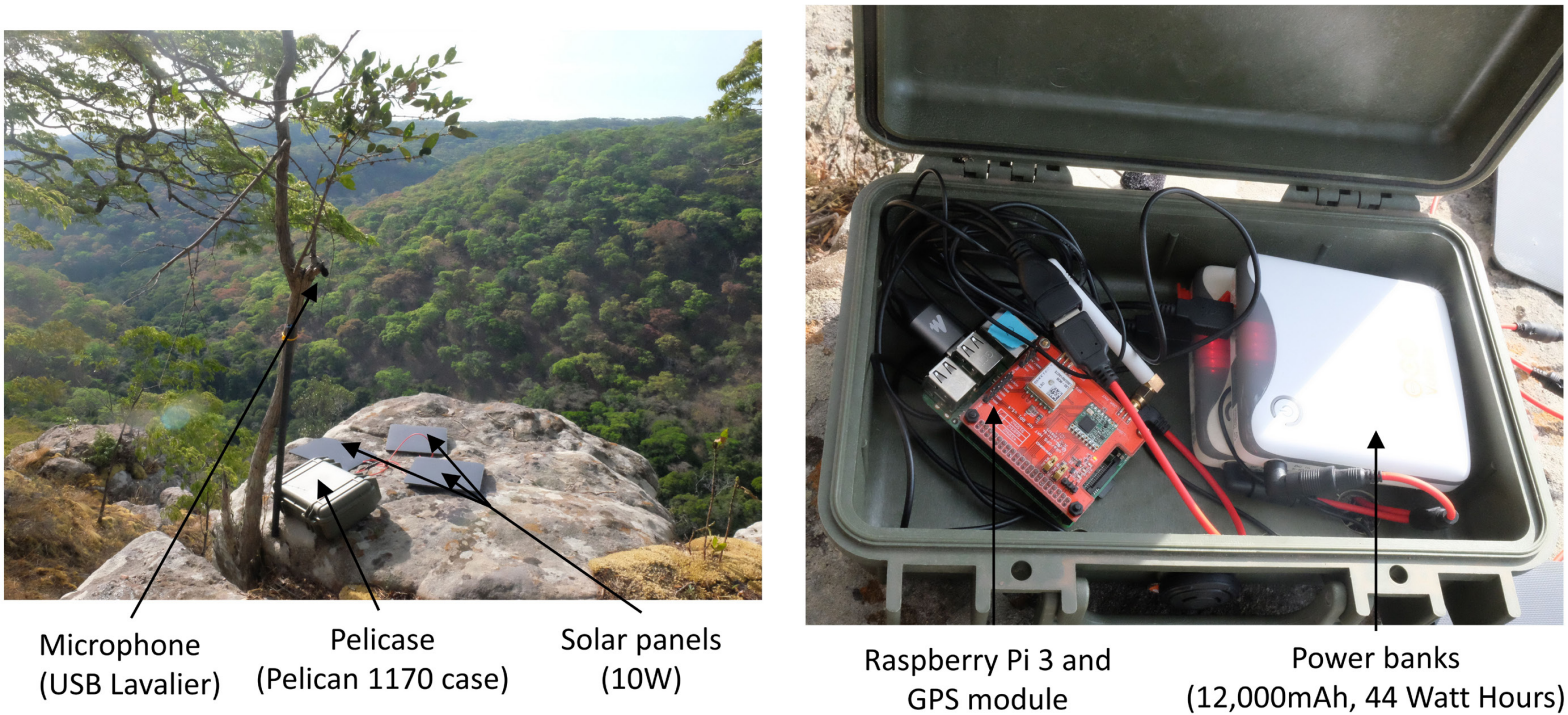
Acoustic localisation system as described in the text

We deployed a passive acoustic monitoring (PAM) system that enables localization of chimpanzee loud calls. The acoustic array consisting of four sensors was deployed around the perimeter of a single valley known to be important for the Issa community during the late dry season (AC personal observation), when we collected the data. Each audio recorder was comprised of a microphone (USB Lavalier omnidirectional) unit integrated with a nano‐computer Raspberry Pi (Raspberry Pi 3 Model B Motherboard); a GPS unit, three 10W solar panels, and two batteries (12,000 mAh, 44 Watt‐Hours) and was protected in a Pelicase (Pelican 1170 Case) (Figure 2). The microphones that we used were USB microphones, so we did not amplify the signal and we used a single microphone per sensor to reduce the cost, complexity, and energy usage of the sensors. Having multiple microphones at each sensor would increase redundancy, but a single microphone is required per sensor to be able to localize the source. The system regularly averaged sensor locations that were determined by the GPS. GPS synchronization is handled using the pulse per second signal from a GPS receiver to convert each sensor node into a Stratum 1 Network Time Protocol (NTP) server. The clocks are automatically adjusted by NTP, and the recordings are synchronized to the clocks on the microcontroller recording device. We placed the sensors on the ground to maximize the energy intake by the solar panels. Sounds were recorded continuously, saved as 30 min audio files at 48 kHz sampling rate (to capture a range of wildlife calls for future studies) in.flac format, and stored on a 32 GB SD card. Each sensor was placed ~500 m from each other to maximize the likelihood of triangulation via detection on multiple sensors, while simultaneously minimizing the likelihood of missing calls. Chimpanzee calls can travel at least 500 m (Alex Piel, unpublished data), so we estimated that the area covered about 1.9 km^2^ by drawing a 500 m buffer around the sensors. We downloaded and saved audio files to an external hard drive every 10 days.

### 2.3 Localization precision and error

**FIGURE 3:**
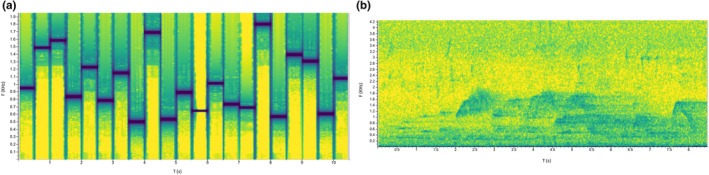
(a) Spectrogram of the tonal sequence used for the playback tests (range 500–1800 Hz), developed from acoustic parameters of a wild chimpanzee pant hoot by Adam Clark Arcadi) and (b) spectrogram of a chimpanzee pant hoot recorded on the ALS

To quantify the error and precision of the system, we conducted two playback studies: a static test and a walking test. For both tests, playback sounds consisted of a tonal sequence (range 500–1800 Hz, Figure 3). This sound sequence was used in place of a pant hoot (the chimpanzee long call—e.g., Goodall, 1986) to minimize disturbance to otherwise xenophobic chimpanzees (Herbinger et al., 2009). We broadcast sounds from 1 m above the ground with a FoxPro Fusion portable loudspeaker (FoxPro Inc., Lewiston, PA, USA) at a mean peak sound pressure level of 103.4 dB (A‐weighting), measured at 1 m from the speaker with a Sound Pressure Level meter (DL7103 Di‐LOG, Manchester, UK). We chose this level to correspond to pant hoots produced by wild individuals (Herbinger et al., 2009). We recorded environmental variables (temperature, wind speed, and relative humidity) with a HOBO weather station (model RX3000) deployed near the research station and about 1 km away from the acoustic array.

The static test consisted of broadcasting repeatedly the tonal sequence at different times of day (6:30, 9:30, 12:30, 15:30, and 18:30), fifty consecutive times at a single location, in the geographic center of the array. The walking test consisted of broadcasting the tonal sequence along line transects, two times each at 30 different locations, sequentially separated by 50 m. We recorded GPS locations with a handheld GPS (Garmin Rino750). In both tests, we faced North when broadcasting the tonal sequence. To quantify the localization precision and error we calculated the Euclidean distance between estimated and true locations.

### 2.4 Validating the localization system with calls from wild chimpanzees

To validate the system with calls from wild chimpanzees, we conducted chimpanzee focal follows. We selected a focal chimpanzee (adult, subadult, or juvenile) each morning and tried to follow the individual for the entire day. We conducted instantaneous focal sampling (Altmann, 1974), with a scan defined as the behavior of the animal recorded every 5 min, when we also collected among other data the location of the individual (GPS). We further noted all‐vocal behavior ad libitum of the focal individual. We then compared the estimated location (see below) of chimpanzee calls recorded by the sensors with the associated locations of the calling chimpanzees determined during focal follows. The minimal distance between the observer and the chimpanzee was 10 m to avoid human–chimpanzee disease transmission, and the GPS location was recorded every 5 min with a handheld GPS.

### 2.5 Time of arrival and sound localization

The time of arrival (TOA) of the sounds was determined at the sub‐second by visualizing the spectrogram with the software Raven, version 1.5 (Bioacoustics Research Program, 2019). We then estimated the sound localizations with the software Sound Finder (Wilson et al., 2014). The software uses the temperature at the time at which the sound is produced to calculate the sound speed following the formula from Wölfel and McDonough (2009). It estimates the location of the sound source by applying the least‐squares solution developed for global positioning systems (Bancroft, 1985), using the TDOA, with the TOA of the sensor reached first set to 0. We defined localization error as the Euclidean distance between estimated and true locations.

### 2.6 Statistical analyses

We conducted all analyses in R v.3.6.1 (R Core Team, 2019). To model the error of the localization (A) as a function of the covariates, we used a linear model. Fixed covariates were temperature (*T*, continuous (°C)), wind (W, continuous (m/s)), number of sensors that detected the sound (S, two levels: 3 or 4 sensors), and vegetation type at the sound source (V, two levels: open or closed). We centered continuous predictors. We ran models with all combinations of predictors and did model averaging among models with ΔAICc < 2.

We tested predictors for collinearity by calculating variation inflation factors (VIF) using the package car (Fox & Weisberg, 2018). Multicollinearity was not present (maximum VIF: *W* = 1.20). We verified model assumptions by plotting residuals versus fitted values and QQ‐plots. We ran a set of models and ranked them by AICc value.

## 3 RESULTS

### 3.1 Localization precision and error

**FIGURE 4:**
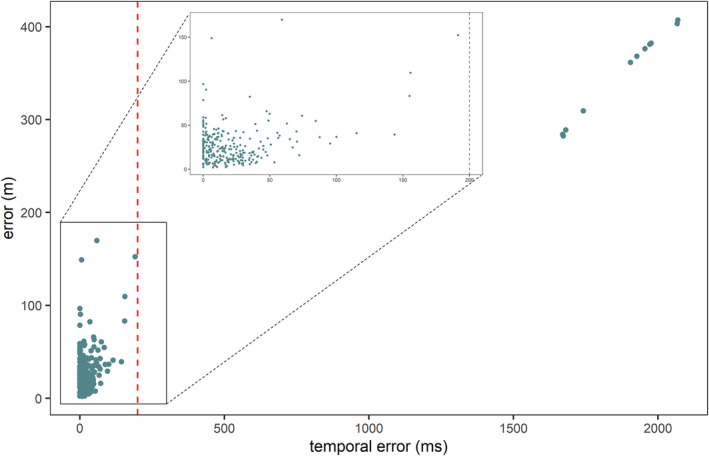
Relationship between the temporal errors associated with the estimated localisations from SoundFinder and the error of the estimated localisations. The red dashed line represents the threshold (200ms) above which the estimated localisation associated

**FIGURE 5:**
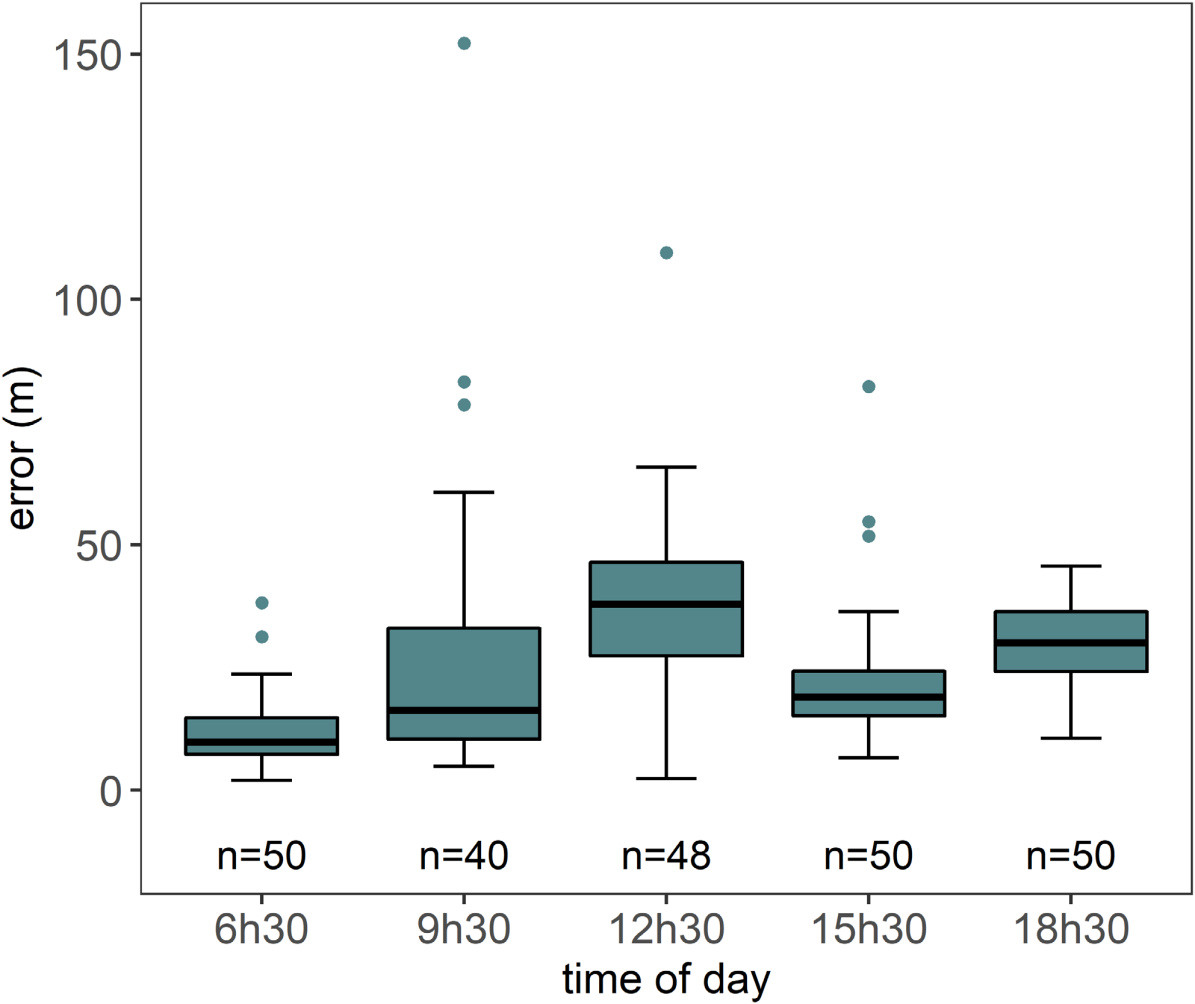
Localisation error at different times of day for the static test

**FIGURE 6:**
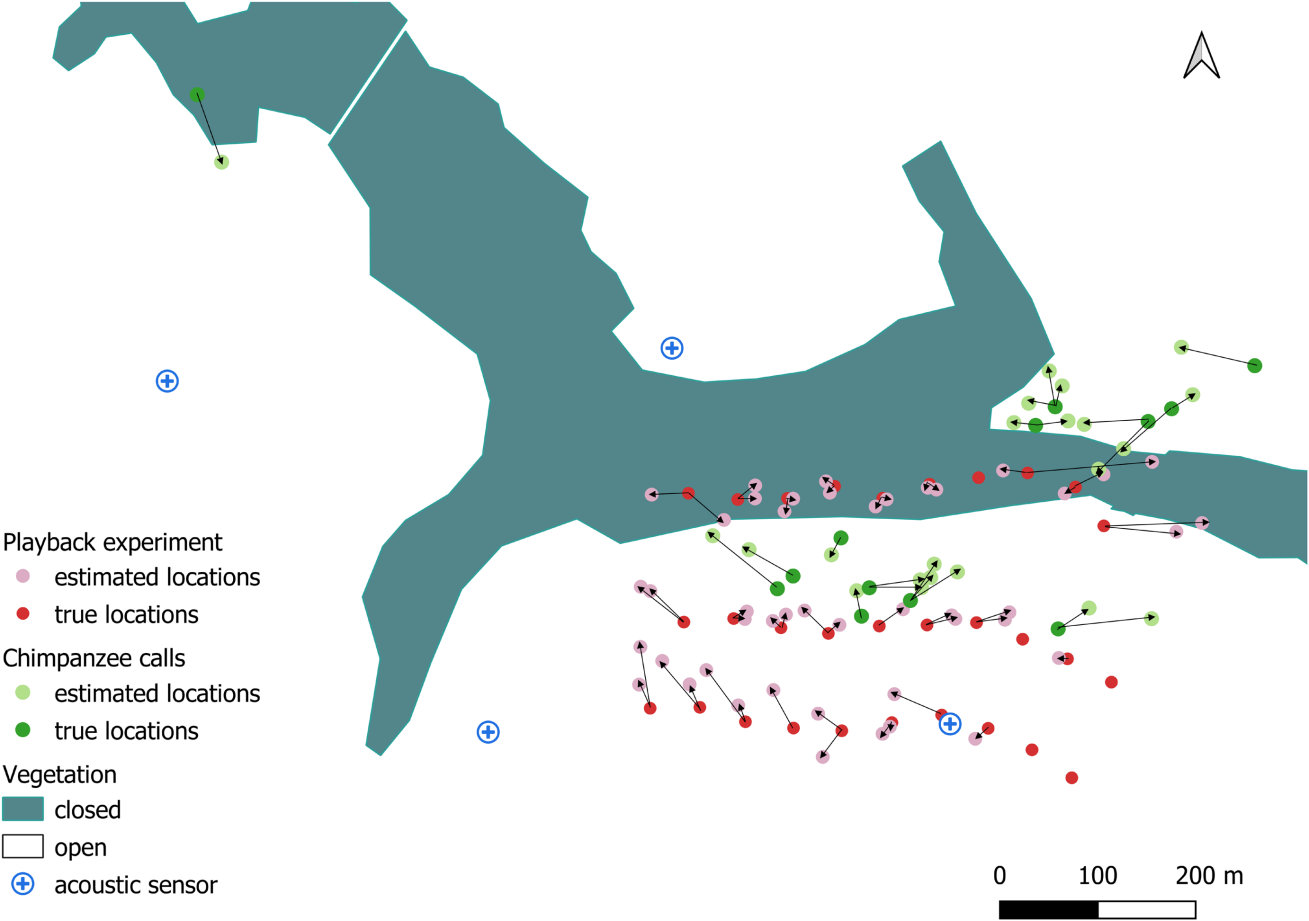
Estimated (pink) and actual (red) locations from the (walking) playback test, and estimated locations by triangulation (light green) of chimpanzees and locations determined with a handheld GPS during parallel focal follows (true locations, dark green); arrows show link between estimated and actual locations

At some locations, the TOA at the acoustic sensors was not possible to calculate because the SNR was too low, or the tonal sequence was only partially recorded. From the 30 locations tested twice on the walking test, we localized 45 of 60 (75%) sounds. From the 250 possible localizations for the static test, we successfully localized 249 sounds (99%). Sound Finder calculates the error of the estimated locations, defined as a temporal error. Similar to Papin et al. (2018), to establish a threshold above which the estimated temporal error associated with the estimated location is considered unreliable, we examined the relationship between localization error and temporal error (Figure 4). Based on these results, we set the threshold to 200 ms, subsequently excluding all estimated locations associated with a temporal error superior to 200 ms. This resulted in 238 estimated locations for the static test (Figure 5) and 44 estimated locations for the walking test (Figure 6). The mean error for all localized sounds was 27 ± 21.8 m [range 2.03–169.8 m, *N* = 282]. Localization was the most precise and less prone to error at 6:30 h, the most prone to error at 12:30 h and the least precise at 9:30 h (Figure 5).

### 3.2 Factors influencing localization error

We did model averaging among models with ΔAICc < 2 (Table 1). The significant effects in the best‐averaged model are temperature, vegetation, and wind (Table 2). Localization error increases as temperature increases and wind speed decreases. Furthermore, localization was less prone to error in open vegetation compared to closed vegetation (Table 2). During the tests, the temperature ranged from 22.3 to 29.8°C (mean 25.5°C) and wind speed ranged from 0.5 to 3 m/s (mean 1.62 m/s).

**TABLE 1:** Model selection. Temperature (*T*), wind (W), number of sensors that detected the sound (S, 3 or 4 sensors), and vegetation type at the sound source (V, open or closed)

Model	df	logLiK	AICc	Delta	Weight
A ~ T + V + W	5	−1049.687	2109.6	0.00	0.656
A ~ T + V + W + S	6	−1049.527	2111.4	1.77	0.270
A ~ T + W	4	−1053.335	2114.8	5.22	0.048
A ~ T + W + S	5	−1052.956	2116.1	6.54	0.025

**TABLE 2:** Outcome of a linear model investigating the effect of temperature, vegetation type at the sound source, wind, and number of sensors that detected the sound on localization error for the averaged best two models

Predictors	Parameter estimate			
	Estimate	*SE*	*z* Value	Pr(>|*z*|)
Intercept	24.477	1.495	16.304	<2e−16***
Temperature	1.488	0.324	4.498	6.9e−06***
Vegetation (open)	18.538	6.9383	2.659	7.83e−03**
Wind	−5.2419	0.999	5.219	2.0e−07***
Sensor	−1.378	2.452	0.558	.577

*p < .05; **p < .01; ***p < .001.

### 3.3 Validating the localization system with chimpanzee calls

We estimated the locations of 22 chimpanzee calls (Figure 6). The mean error was 51.2 ± 20.6 m [range 19.9–96.04 m].

## 4 DISCUSSION

In this study, we sought to demonstrate, as a proof of concept, that a custom‐made acoustic array composed of four sensors could localize wild chimpanzees. The array enables sound localization in a mountainous environment with heterogeneous vegetation that makes sound propagation unpredictable. Using data conducted from a playback study, we found that the mean localization error was 27 ± 21.8 m. To empirically validate the system, we also successfully localized wild chimpanzee calls, applying this system under natural conditions. We compare the error of this system with previous ALSs for terrestrial species described in the literature. We explore the behavioral and conservation applications of this approach to the study of wild chimpanzees and more broadly other loud calling terrestrial species and conclude with a discussion of the limitations of the current system.

### 4.1 Error of the localization system

We found that the error of the ALS was similar to those figures reported from other studies targeting terrestrial mammals. These systems covered areas that ranged from a few hundreds to thousands of m^2^, reflecting the ranges of the targeted species. Other systems that target birds and frogs localized animals with less error, in some cases sub‐meter. These arrays were composed of spatially closer acoustic sensors (<50 m) offering far less spatial coverage (Table 3).

**TABLE 3:** Previously described terrestrial acoustic localization systems and reported error

Target species	Acoustic array	Error	Reference
Cape buffalo (Syncerus caffer), chacma baboon (Papio ursinus), and spotted hyena (Crocuta crocuta)	Four CARACAL stations at 500 m intervals	Within 70 m	Wijers et al. (2019)
Chimpanzee (Pan troglodytes)	Four custom‐made recorders at 543.7 ± 163.8 m intervals	27 ± 21.8 m	This study
Elephant (Elephas maximus)	Four Audio Technica recorders	30 m	Dissanayake et al. (2018)
Orangutan (Pongo pygmaeus wurmbii)	20 SM2 (Wildlife Acoustics) recorders at 500 m intervals	58m ± 7.2 m	Spillmann et al. (2015)
Wolf (Canis lupus)	20 SM3 (Wildlife Acoustics) recorders at 1 km intervals	167 ± 308 m	Papin et al. (2018)
Wolf (Canis lupus)	Five SM3 (Wildlife Acoustics) at 1–3 km intervals	20 m	Kershenbaum et al. (2019)
Rufous‐and‐white wren (Thryothorus rufalbus)	Eight microphones at 75.2 ± 2.6 m intervals	2.82 ± 0.26 m	Mennill et al. (2006)
Antbird (Formicarius moniliger)	Eight nodes (each node contains four microphones) at 39 m intervals	0.199 ± 0.064 m for playbacks and 0.445 ± 0.500 m for wild bird songs	Collier et al. (2010)
Different bird and frog species	Four SM2 (Wildlife Acoustics) recorders at 25 or 50 m intervals	1.87 ± 0.13 m	Mennill et al. (2012)

Environmental variables such as temperature and wind speed influence sound behavior, for example, sound attenuation (Harris, 1966) and consequently, localization error. Sounds were less prone to error and more precisely localized in the early morning (6:30 h), when the temperature was the lowest and wind speed is the highest. This is the same period when chimpanzees are the most vocally active (Piel, 2018; Wilson et al., 2007). Conversely, localization error was the highest at 12:30 h, when the temperature is the highest. It could be that temperature and wind speed interact to produce the most optimum conditions for sound to be localized. We were indeed surprised by the low wind‐high error association, as that is contrary to well‐established relationships between wind and sound propagation. We did not account for wind direction, however, and thus our results could reveal that downwind are helping sounds avoid landscape features (e.g., trees) that otherwise attenuate sounds (Trikootam & Hornikx, 2019). Subsequent testing on propagation and wind direction will be critical to resolving this.

### 4.2 Behavioral applications

Despite over a half‐century of research into wild chimpanzees (Boesch et al., 2019; Nakamura et al., 2015; Pusey et al., 2007) and in the deployments of PAM for wildlife in other systems (Marques et al., 2013; Spiesberger & Fristrup, 1990; Tavolga, 2012), only a few studies have deployed this tool with wild apes, and only one, besides the current study, evaluated its localization accuracy (Spillmann et al., 2015, Table 3). Acoustically localizing chimpanzees offer multiple benefits to behavioral study of habituated and unhabituated individuals. First, the resulting data can improve our understanding of social dynamics. Similar to other species such as elephants (*Loxodonta africana*) (Leighty et al., 2008), spotted hyenas (*Crocuta crocuta*), (e.g., Theis et al., 2007), bottlenose dolphins (*Tursiops truncates*) (Janik & Slater, 1998) chimpanzees exhibit a fission–fusion structure (Fedurek et al., 2014). They form ephemeral sub‐parties that change in size and composition throughout the day. So far, little is known about how chimpanzees coordinate sub‐group reunions, more specifically at their nesting sites, and maintain cohesion within the community (Lehmann & Boesch, 2004). This is especially the case of savanna‐mosaic dwelling chimpanzees, who live at a density up to 10 times lower than their forest‐dwelling counter‐parts—for example, 0.56 ind./km^2^ at Issa, Tanzania (Anne‐Sophie Crunchant, unpublished data) versus 6.8 ind./km^2^ at Budongo, Uganda (Newton‐Fisher, 2003)—and cover territory far larger—for example, ≥55 km^2^ at Issa, Tanzania versus 6.8 km^2^ at Budongo, Uganda (Newton‐Fisher, 2003). Only two studies have attempted multiple, simultaneous focal follows (Eckhardt et al., 2015; Uhlenbroek, 1996), despite our knowledge of the importance of vocalizations for spatially separated callers (Fedurek et al., 2014; Gruber & Zuberbühler, 2013). One means of overcoming the logistical demands of multiple follows is using an ALS, consequently detecting (and potentially monitoring) caller presence in space and time, which has been done in the marine environment. For instance, dolphins (*Delphinus delphis*) can be tracked via their whistles that can propagate over multiple kilometres omnidirectionally (Wiggins et al., 2013). The authors showed with whistle localization that dolphins were more widely spread and travelled more slowly at the beginning of the night in contrast to daytime and hypothesized that it was associated with foraging behaviur. Previous work has revealed that under optimal sound prorogation conditions, playback calls (and by implication pant hoots) can be recorded from >3 km from their origin (Piel, 2014), suggesting a larger array with only a modest increase in sensors could monitor social dynamics over a vast area over which chimpanzees range.

To track chimpanzee movements over time, we need caller individual identification. Acoustic detectors of chimpanzee calls are in development (Heinicke et al., 2015). Individual identification remains complicated, however, due to the high intra‐ and inter‐caller variability of chimpanzee calls and their large vocal repertoire highly graded (call types are difficult to categorize) (Crockford, 2019; Mitani et al., 1996). Call combinations and the chorusing effect, where multiple individuals vocalize simultaneously add another level of complexity to developing a call detector. Vocal individuality has been found in other terrestrial species, such as tigers (*Panthera tigris*) (Ji et al., 2013), orangutans (*Pongo pygmaeus wurmbii*) (Spillmann et al., 2016), and gibbons (*Hylobates muelleri*) (Clink et al., 2018). With new machine learning pipelines, we are confident that vocal individuality and later individual identification detectors will be developed for chimpanzees.

A second behavioral application of ALS is to accelerate the habituation process, one that is especially time‐intensive with chimpanzees—for example, ~5–7 years, Taï Forest, Côte d’Ivoire (Bertolani & Boesch, 2008). Historically, researchers attempted to habituate chimpanzees to human presence by provisioning them with food (Goodall, 1986; Nishida, 2011; Wrangham, 1974). However, this method increases disease risk for wild animals and subsequently modifies natural behavior patterns. It is now universally discouraged (Williamson & Feistner, 2003; Wrangham, 1974). Instead of provisioning, to find unhabituated animals, often researchers listen to specific locations (e.g., at the top and junction of different valleys) for chimpanzee loud calls or wait at key feeding trees (Williamson & Feistner, 2003). If researchers had access to chimpanzee caller locations—especially when looking for parties, the efficiency of search efforts would be dramatically improved. It is nearly impossible to quantify the extent of this improvement, but Sommer et al. (2004) report seeing an individual chimpanzee on average once every 22.8 days during the two first years of habituation at the Gashaka Gumti National Park, Nigeria. Even though chimpanzees could be outside of sensor range, integrating traditional search efforts with an ALS would likely improve search efficiency, especially with (near) real‐time data transmission.

### 4.3 Conservation applications

Density is a critical parameter for species monitoring. New methods combining PAM and spatially explicit capture‐recapture models have been developed to estimate animal density (Dawson & Efford, 2009; Efford et al., 2009; Measey et al., 2017; Stevenson et al., 2015). The addition of auxiliary data, such as TDOA or signal strength provides more accurate information on the distance between the caller and the acoustic sensor, in turn allowing more precise detection functions and density estimation (Stevenson et al., 2015), and will thus benefit monitoring efforts.

The ALS also enables key resources localization, such as the presence of chimpanzees at fruiting trees. Chimpanzees produce calls with a different acoustic structure (e.g., peak frequency and call duration) as a function of the food patch size or tree species (Fedurek et al., 2014; Kalan et al., 2015; Slocombe & Zuberbühler, 2006). Being able to locate such feeding trees via the calls produced by chimpanzees will first help provide a broader picture of their feeding ecology and second further aid habituation efforts (see above). Similarly, an ALS will also enable researchers to identify chimpanzee nesting sites. Locating chimpanzees at their nesting sites and thus indirectly locating fresh nests with the ALS will benefit conservation by allowing researchers to collect, for example, fresh fecal samples that can reveal population dynamics (Schwartz et al., 2007) and are useful for health monitoring (Gilardi et al., 2015).

Finally, poaching and deforestation are the two main threats to great apes (Estrada et al., 2017). Besides detecting animals, ALSs can also indirectly help species conservation by revealing poachers via gunshot sounds (Wijers et al., 2019) or locating illegal logging via chainsaw sounds (Andrei et al., 2015). A few platforms have recently been field‐tested but are not widely used yet. For instance, CARACAL is low‐cost hardware (~£150 per unit) and software able to extract and localize gunshots at an average accuracy of 33.2 m with an array of seven stations composed of four microphones (Wijers et al., 2019). ALS can thus be used as a law enforcement tool to assist conservationists and prevent animal poaching or deforestation.

### 4.4 Limitations

There are three primary limitations of the current study. First, we did not consider the GPS accuracy. GPS locations at each sensor were averaged but given that all sensors were stationary, we suspect minimal errors due to GPS sensor values. However, the exact accuracy of the handheld device used to measure ground truth is unknown.

Second, we were not able to capture microhabitat (environmental) variation, which may have affected sound propagation (Rodriguez et al., 2014; Röhr & Juncá, 2013). In the current study, we used the weather data from a centrally located weather station, >1000 m from the nearest sensor. More spatially explicit weather data would be useful. This is especially important for some variables like wind speed, which is known to vary significantly, especially in valley systems (Lihoreau et al., 2006; Renterghem et al., 2007). Furthermore, we did not evaluate the effect of the caller position, that is, whether they were terrestrial or arboreal. Previous studies have shown that caller height and the frequency at which they vocalize have an impact on sound transmission. Lower frequencies propagate further when the animal vocalizes higher than 1 m above the ground, due to an increase in the effective area by reducing the attenuating effect of soft ground (Forrest, 1994; Marten & Marler, 1977; Parris, 2002). Similarly, we did not assess the effect of ambient noise level on localization error. The TDOA estimation error depends on the SNR (Urazghildiiev & Clark, 2013). It has been shown that sound level increases during early evenings (Piel, 2014), which could explain why localization error was lower at 18:30 h compared to 6:30 h, for similar temperatures and higher wind speed early morning. Dawn chorus is a well‐studied phenomenon exhibited by multiple species and has been studied especially on birds (reviewed in Gil & Llusia, 2020). Among multiple hypotheses for this behavior such as advertising territory boundaries and social dynamics, the hypothesis of a better sound transmission at dawn has been evocated (Henwood & Fabrick, 1979) but is controversial (Gil & Llusia, 2020).

Lastly, we conducted manual localization analyses. TDOA is often estimated by pairwise cross‐correlations of the sound waveforms or spectrograms (Harlow et al., 2013; Mennill et al., 2006; Spillmann et al., 2015). Similar to Papin et al. (2018) and Kershenbaum et al. (2019), we manually estimated TDOA from the spectrograms due to the low SNR of some of the playbacks or chimpanzee calls. If manual analyses allow for decreasing the probability of missing a call, they can also be prone to errors. Indeed, TOA needs to be measured very accurately (onset can vary by <1 ms) and manual measurement can lower localization accuracy (Rhinehart et al., 2020). Furthermore, such analyses are time intensive.

## 5 CONCLUSION

In this study, we have demonstrated the performance of a low‐cost custom‐made ALS for chimpanzee localization. The ALS powered by a solar system can be deployed for long periods (only limited by storage capacity), and the recording script is easily modifiable in Python, for example, adding a recording schedule, changing the recorded frequency, or file length. Like other PAM systems, it allows for the study of conspicuous or even cryptic animals without disturbing them. With recent technological advances, devices are increasingly robust and affordable. Despite the current challenges to automating data analysis, improvements in automatic call detection are promising, and we anticipate that PAM and ALS will become more frequently deployed tools for loud calling terrestrial species monitoring.

## CONFLICT OF INTEREST

The authors declare no conflicts of interest.

## AUTHOR CONTRIBUTIONS

**Anne‐Sophie Crunchant:** Conceptualization (equal); Formal analysis (lead); Funding acquisition (equal); Methodology (equal); Visualization (lead); Writing – original draft (lead); Writing – review & editing (lead). **Jason T. Isaacs:** Conceptualization (equal); Funding acquisition (equal); Methodology (equal); Software (lead); Supervision (equal); Writing – review & editing (equal). **Alex K. Piel:** Conceptualization (equal); Funding acquisition (equal); Project administration (lead); Supervision (equal); Writing – review & editing (equal).

## Data Availability

Audio files are available from the Figshare data repository (https://doi.org/10.6084/m9.figshare.13140338).

## References

[ece38902-bib-0001] Altmann, J. (1974). Observational study of behavior: Sampling methods. Behaviour, 49(3), 227–267. 10.1080/14794802.2011.585831 4597405

[ece38902-bib-0002] Andrei, V. , Cucu, H. , & Petrică, L. (2015). Considerations on developing a chainsaw intrusion detection and localization system for preventing unauthorized logging. Journal of Electrical and Electronic Engineering, 3(6), 202–207. 10.11648/j.jeee.20150306.15

[ece38902-bib-0003] Bancroft, S. (1985). An algebraic solution of the GPS equations. IEEE Transactions on Aerospace and Electronic Systems, AES‐21(1), 56–59. 10.1109/TAES.1985.310538

[ece38902-bib-0004] Bertolani, P. , & Boesch, C. (2008). Habituation of wild chimpanzees (*Pan troglodytes*) of the south group at Taï Forest, Côte d’Ivoire: Empirical measure of progress. Folia Primatologica, 79, 162–171. 10.1159/000111720 18057910

[ece38902-bib-0005] Bioacoustics Research Program (2019). Raven Pro: Interactive sound analysis software (version 1.5). The Cornell Lab of Ornithology.

[ece38902-bib-0006] Blumstein, D. T. , Mennill, D. J. , Clemins, P. , Girod, L. , Yao, K. , Patricelli, G. , Deppe, J. L. , Krakauer, A. H. , Clark, C. , Cortopassi, K. A. , Hanser, S. F. , McCowan, B. , Ali, A. M. , & Kirschel, A. N. G. (2011). Acoustic monitoring in terrestrial environments using microphone arrays: Applications, technological considerations and prospectus. Journal of Applied Ecology, 48(3), 758–767. 10.1111/j.1365-2664.2011.01993.x

[ece38902-bib-0007] Boesch, C. , Wittig, R. , Crockford, C. , Vigilant, L. , Deschner, T. , & Leendertz, F. (Eds.). (2019). The chimpanzees of the Taï Forest: 40 years of research. Cambridge University Press.

[ece38902-bib-0008] Clink, D. J. , Crofoot, M. C. , & Marshall, A. J. (2018). Application of a semi‐automated vocal fingerprinting approach to monitor Bornean gibbon females in an experimentally fragmented landscape in Sabah, Malaysia. Bioacoustics, 28, 193–209. 10.1080/09524622.2018.1426042

[ece38902-bib-0009] Collier, T. C. , Kirschel, A. N. G. , & Taylor, C. E. (2010). Acoustic localization of antbirds in a Mexican rainforest using a wireless sensor network. The Journal of the Acoustical Society of America, 128, 182–189. 10.1121/1.3425729 20649213

[ece38902-bib-0010] Crockford, C. (2019). Why does the chimpanzee vocal repertoire remain poorly understood and what can be done about it? In C. Boesch & R. Wittig (Eds.), The Chimpanzees of the Taï Forest (pp. 394–409). Cambridge University Press. 10.1017/9781108674218.025

[ece38902-bib-0011] Crunchant, A.‐S. , Borchers, D. , Kühl, H. , & Piel, A. (2020). Listening and watching: Do camera traps or acoustic sensors more efficiently detect wild chimpanzees in an open habitat? Methods in Ecology and Evolution, 11(4), 1–11. 10.1111/2041-210X.13362

[ece38902-bib-0012] Crunchant, A. S. , Stewart, F. A. , & Piel, A. K. (2021). Vocal communication in wild chimpanzees: A call rate study. PeerJ, 9, 1–19. 10.7717/peerj.12326 PMC8532989 34721995

[ece38902-bib-0013] Dawson, D. K. , & Efford, M. G. (2009). Bird population density estimated from acoustic signals. Journal of Applied Ecology, 46, 1201–1209. 10.1111/j.1365-2664.2009.01731.x

[ece38902-bib-0014] Dissanayake, C. M. , Kotagiri, R. , Halgamuge, M. N. , & Moran, B. (2018). Improving accuracy of elephant localization using sound probes. Applied Acoustics, 129, 92–103. 10.1016/j.apacoust.2017.07.007

[ece38902-bib-0015] Eckhardt, N. , Polansky, L. , & Boesch, C. (2015). Spatial cohesion of adult male chimpanzees (*Pan troglodytes verus*) in Taï National Park, Côte d’Ivoire. American Journal of Primatology, 77, 125–134. 10.1002/ajp.22316 25256306

[ece38902-bib-0016] Efford, M. G. , Dawson, D. K. , & Borchers, D. L. (2009). Population density estimated from locations of individuals on a passive detector array. Ecology, 90(10), 2676–2682. 10.1890/08-1735.1 19886477

[ece38902-bib-0017] Estrada, A. , Garber, P. A. , Rylands, A. B. , Roos, C. , Fernandez‐Duque, E. , Di Fiore, A. , Nekaris, K.‐I. , Nijman, V. , Heymann, E. W. , Lambert, J. E. , Rovero, F. , Barelli, C. , Setchell, J. M. , Gillespie, T. R. , Mittermeier, R. A. , Arregoitia, L. V. , de Guinea, M. , Gouveia, S. , Dobrovolski, R. , … Li, B. (2017). Impending extinction crisis of the world’s primates: Why primates matter. Science Advances, 3(1), e1600946. 10.1126/sciadv.1600946 28116351 PMC5242557

[ece38902-bib-0018] Fedurek, P. , Donnellan, E. , & Slocombe, K. E. (2014). Social and ecological correlates of long‐distance pant hoot calls in male chimpanzees. Behavioral Ecology and Sociobiology, 68, 1345–1355. 10.1007/s00265-014-1745-4

[ece38902-bib-0019] Fitzsimmons, L. P. , Foote, J. R. , Ratcliffe, L. M. , & Mennill, D. J. (2008). Eavesdropping and communication networks revealed through playback and an acoustic location system. Behavioral Ecology, 19(4), 824–829. 10.1093/beheco/arn036

[ece38902-bib-0020] Forrest, T. G. (1994). From sender to receiver: Propagation and environmental effects on acoustic signals. American Zoologist, 34, 644–654. 10.1093/icb/34.6.644

[ece38902-bib-0021] Fox, J. , & Weisberg, S. (2018). An R companion to applied regression. Sage publications.

[ece38902-bib-0022] Gil, D. , & Llusia, D. (2020). The bird dawn chorus revisited. In T. Aubin , & N. Mathevon (Eds.), Coding strategies in vertebrate acoustic communication (pp. 45–90). Springer. 10.1007/978-3-030-39200-0_3

[ece38902-bib-0023] Gilardi, K. V. , Gillespie, T. R. , Leendertz, F. H. , Macfie, E. J. , Travis, D. A. , Whittier, C. A. , & Williamson, E. A. (2015). Best practice guidelines for health monitoring and disease control in great ape populations. IUCN/SSC Primate Specialist Group. 10.2305/iucn.ch.2015.ssc-op.56.en

[ece38902-bib-0024] Goodall, J. (1986). The chimpanzees of Gombe: Patterns of behavior. Harvard University Press.

[ece38902-bib-0025] Gruber, T. , & Zuberbühler, K. (2013). Vocal recruitment for joint travel in wild chimpanzees. PLoS One, 8(9), e76073. 10.1371/journal.pone.0076073 24086688 PMC3783376

[ece38902-bib-0026] Harlow, Z. , Collier, T. , Burkholder, V. , & Taylor, C. E. (2013). Acoustic 3D localization of a tropical songbird. In 2013 IEEE China Summit and International Conference on Signal and Information Processing (ChinaSIP), 220–224. IEEE. 10.1109/ChinaSIP.2013.6625332

[ece38902-bib-0027] Harris, C. M. (1966). Absorption of sound in air versus humidity and temperature. Journal of the Acoustical Society of America, 40, 148–159. 10.1121/1.1910031

[ece38902-bib-0028] Hedwig, D. , DeBellis, M. , & Wrege, P. H. (2018). Not so far: Attenuation of low‐frequency vocalizations in a rainforest environment suggests limited acoustic mediation of social interaction in African forest elephants. Behavioral Ecology and Sociobiology, 72(3), 33. 10.1007/s00265-018-2451-4

[ece38902-bib-0029] Heinicke, S. , Kalan, A. K. , Wagner, O. J. J. , Mundry, R. , Lukashevich, H. , & Kühl, H. S. (2015). Assessing the performance of a semi‐automated acoustic monitoring system for primates. Methods in Ecology and Evolution, 6, 753–763. 10.1111/2041-210x.12384

[ece38902-bib-0030] Henwood, K. , & Fabrick, A. (1979). A quantitative analysis of the dawn chorus: Temporal selection for communicatory optimization. The American Naturalist, 114(2), 260–274. 10.1086/283473

[ece38902-bib-0031] Herbinger, I. , Papworth, S. , Boesch, C. , & Zuberbühler, K. (2009). Vocal, gestural and locomotor responses of wild chimpanzees to familiar and unfamiliar intruders: A playback study. Animal Behaviour, 78(6), 1389–1396. 10.1016/j.anbehav.2009.09.010

[ece38902-bib-0032] Janik, V. M. , & Slater, P. J. B. (1998). Context‐specific use suggests that bottlenose dolphin signature whistles are cohesion calls. Animal Behaviour, 56, 829–838. 10.1006/anbe.1998.0881 9790693

[ece38902-bib-0033] Ji, A. , Johnson, M. T. , Walsh, E. J. , McGee, J. , & Armstrong, D. L. (2013). Discrimination of individual tigers (*Panthera tigris*) from long distance roars. The Journal of the Acoustical Society of America, 133(3), 1762–1769. 10.1121/1.4789936 23464045

[ece38902-bib-0034] Kalan, A. K. , Mundry, R. , & Boesch, C. (2015). Wild chimpanzees modify food call structure with respect to tree size for a particular fruit species. Animal Behaviour, 101, 1–9. 10.1016/j.anbehav.2014.12.011

[ece38902-bib-0035] Kays, R. , Crofoot, M. C. , Jetz, W. , & Wikelski, M. (2015). Terrestrial animal tracking as an eye on life and planet. Science, 348(6240), aaa2478. 10.1126/science.aaa2478 26068858

[ece38902-bib-0036] Kershenbaum, A. , Owens, J. L. , & Waller, S. (2019). Tracking cryptic animals using acoustic multilateration: A system for long‐range wolf detection. The Journal of the Acoustical Society of America, 145(3), 1619–1628. 10.1121/1.5092973 31067959

[ece38902-bib-0037] Lehmann, J. , & Boesch, C. (2004). To fission or to fusion: Effects of community size on wild chimpanzee (*Pan troglodytes verus*) social organisation. Behavioral Ecology and Sociobiology, 56(3), 207–216. 10.1007/s00265-004-0781-x

[ece38902-bib-0038] Leighty, K. A. , Soltis, J. , Wesolek, C. M. , & Savage, A. (2008). Rumble vocalizations mediate interpartner distance in African elephants, *Loxodonta africana* . Animal Behaviour, 76, 1601–1608. 10.1016/j.anbehav.2008.06.022

[ece38902-bib-0039] Lihoreau, B. , Gauvreau, B. , Bérengier, M. , Blanc‐Benon, P. , & Calmet, I. (2006). Outdoor sound propagation modeling in realistic environments: Application of coupled parabolic and atmospheric models. The Journal of the Acoustical Society of America, 120(1), 110–119. 10.1121/1.2204455

[ece38902-bib-0040] Marler, P. , & Hobbett, L. (1975). Individuality in a long‐range vocalization of wild chimpanzees. Zeitschrift Für Tierpsychologie, 38, 97–109. 10.1111/j.1439-0310.1975.tb01994.x 809940

[ece38902-bib-0041] Marques, T. A. , Thomas, L. , Martin, S. W. , Mellinger, D. K. , Ward, J. A. , Moretti, D. J. , Harris, D. , & Tyack, P. L. (2013). Estimating animal population density using passive acoustics. Biological Reviews, 88, 287–309. 10.1111/brv.12001 23190144 PMC3743169

[ece38902-bib-0042] Marten, K. , & Marler, P. (1977). Sound transmission and its significance for animal vocalization: I. Temperate habitats. Behavioral Ecology and Sociobiology, 2(3), 271–290. 10.1007/BF00299740

[ece38902-bib-0043] Measey, G. J. , Stevenson, B. C. , Scott, T. , Altwegg, R. , & Borchers, D. L. (2017). Counting chirps: Acoustic monitoring of cryptic frogs. Journal of Applied Ecology, 54, 894–902. 10.1111/1365-2664.12810

[ece38902-bib-0044] Mennill, D. J. , Battiston, M. , Wilson, D. R. , Foote, J. R. , & Doucet, S. M. (2012). Field test of an affordable, portable, wireless microphone array for spatial monitoring of animal ecology and behaviour. Methods in Ecology and Evolution, 3(4), 704–712. 10.1111/j.2041-210X.2012.00209.x

[ece38902-bib-0045] Mennill, D. J. , Burt, J. M. , Fristrup, K. M. , & Vehrencamp, S. L. (2006). Accuracy of an acoustic location system for monitoring the position of duetting songbirds in tropical forest. The Journal of the Acoustical Society of America, 119, 2832–2839. 10.1121/1.2184988 16708941 PMC2247711

[ece38902-bib-0046] Millspaugh, J. J. , & Marzluff, J. M. (2001). Radio‐tracking and animal populations: Past trends and future needs. In J. J. Millspaugh & J. M. Marzluff (Eds.), Radio tracking and animal populations (pp. 383–393). Academic Press. 10.1016/B978-012497781-5/50016-5

[ece38902-bib-0047] Mitani, J. C. , Gros‐Louis, J. , & Macedonia, J. M. (1996). Selection for acoustic individuality within the vocal repertoire of wild chimpanzees. International Journal of Primatology, 17(4), 569–583. 10.1007/BF02735192

[ece38902-bib-0048] Nakamura, M. , Hosaka, K. , Itoh, N. , Zamma, K. (Eds.). (2015). Mahale chimpanzees: 50 years of research. Cambridge University Press.

[ece38902-bib-0049] Newton‐Fisher, N. E. (2003). The home range of the Sonso community of chimpanzees from the Budongo Forest, Uganda. African Journal of Ecology, 41, 150–156. 10.1046/j.1365-2028.2003.00408.x

[ece38902-bib-0050] Nishida, T. (2011). Chimpanzees of the lakeshore: Natural history and culture at Mahale. Cambridge University Press.

[ece38902-bib-0051] Papin, M. , Pichenot, J. , Guérold, F. , & Germain, E. (2018). Acoustic localization at large scales: A promising method for grey wolf monitoring. Frontiers in Zoology, 15, 1–10. 10.1186/s12983-018-0260-2 29681989 PMC5897954

[ece38902-bib-0052] Parris, K. M. (2002). More bang for your buck: The effect of caller position, habitat and chorus noise on the efficiency of calling in the spring peeper. Ecological Modelling, 156(2–3), 213–224. 10.1016/S0304-3800(02)00170-9

[ece38902-bib-0053] Piel, A. K. (2014). Savanna sounds: Using remote acoustic sensing to study spatiotemporal patterns in wild chimpanzee loud vocalizations in the Issa Valley. University of California, San Diego. 10.1300/J122v22n03_06

[ece38902-bib-0054] Piel, A. K. (2018). Temporal patterns of chimpanzee loud calls in the Issa Valley, Tanzania: Evidence of nocturnal acoustic behavior in wild chimpanzees. American Journal of Physical Anthropology, 166(3), 530–540. 10.1002/ajpa.23609 29989161

[ece38902-bib-0055] Pusey, A. E. , Pintea, L. , Wilson, M. L. , Kamenya, S. , & Goodall, J. (2007). The contribution of long‐term research at Gombe National Park to chimpanzee conservation. Conservation Biology, 21(3), 623–634. 10.1111/j.1523-1739.2007.00704.x 17531041

[ece38902-bib-0056] R Core Team . (2019). R: A language and environment for statistical computing. R Foundation for Statistical Computing. https://www.r‐project.org

[ece38902-bib-0057] Renterghem, T. V. , Botteldooren, D. , & Lercher, P. (2007). Comparison of measurements and predictions of sound propagation in a valley‐slope configuration in an inhomogeneous atmosphere. The Journal of the Acoustical Society of America, 121(5), 2522–2533. 10.1121/1.2717765 17550151

[ece38902-bib-0058] Rhinehart, T. A. , Chronister, L. M. , Devlin, T. , & Kitzes, J. (2020). Acoustic localization of terrestrial wildlife: Current practices and future opportunities. Ecology and Evolution, 1–25, 10.1002/ece3.6216 PMC7381569 32724552

[ece38902-bib-0059] Rodriguez, A. , Gasc, A. , Pavoine, S. , Grandcolas, P. , Gaucher, P. , & Sueur, J. (2014). Temporal and spatial variability of animal sound within a neotropical forest. Ecological Informatics, 21, 133–143. 10.1016/j.ecoinf.2013.12.006

[ece38902-bib-0060] Röhr, D. L. , & Juncá, F. A. (2013). Micro‐habitat influence on the advertisement call structure and sound propagation efficiency of *Hypsiboas crepitans* (Anura: Hylidae). Journal of Herpetology, 47(4), 549–554. 10.1670/10-210

[ece38902-bib-0061] Schwartz, M. K. , Luikart, G. , & Waples, R. S. (2007). Genetic monitoring as a promising tool for conservation and management. Trends in Ecology and Evolution, 22, 25–33. 10.1016/j.tree.2006.08.009 16962204

[ece38902-bib-0062] Slocombe, K. E. , & Zuberbühler, K. (2006). Food‐associated calls in chimpanzees: Responses to food types or food preferences? Animal Behaviour, 72(5), 989–999. 10.1016/j.anbehav.2006.01.030

[ece38902-bib-0063] Smith, B. R. , Root‐Gutteridge, H. , Butkiewicz, H. , Dassow, A. , Fontaine, A. C. , Markham, A. , Owens, J. , Schindler, L. , Wijers, M. , & Kershenbaum, A. (2021). Acoustic localisation of wildlife with low‐cost equipment: Lower sensitivity, but no loss of precision. Wildlife Research. 10.1071/WR21089

[ece38902-bib-0064] Sommer, V. , Adanu, J. , Faucher, I. , & Fowler, A. (2004). Nigerian chimpanzees (*Pan troglodytes vellerosus*) at Gashaka: Two years of habituation efforts. Folia Primatologica, 75, 295–316. 10.1159/000080208 15486442

[ece38902-bib-0065] Spiesberger, J. L. , & Fristrup, K. M. (1990). Passive localization of calling animals and sensing of their acoustic environment using acoustic tomography. The American Naturalist, 135, 107–153. 10.1086/285035

[ece38902-bib-0066] Spillmann, B. , van Noordwijk, M. A. , Willems, E. P. , Mitra Setia, T. , Wipfli, U. , & van Schaik, C. P. (2015). Validation of an acoustic location system to monitor Bornean orangutan (*Pongo pygmaeus wurmbii*) long calls. American Journal of Primatology, 77(7), 767–776. 10.1002/ajp.22398 25773926

[ece38902-bib-0067] Spillmann, B. , van Schaik, C. P. , Setia, T. M. , & Sadjadi, S. O. (2016). Who shall I say is calling? Validation of a caller recognition procedure in Bornean flanged male orangutan (*Pongo pygmaeus wurmbii*) long calls. Bioacoustics, 26(2), 109–120. 10.1080/09524622.2016.1216802

[ece38902-bib-0068] Stafford, K. M. , Fox, C. G. , & Clark, D. S. (1998). Long‐range acoustic detection and localization of blue whale calls in the northeast Pacific Ocean. The Journal of the Acoustical Society of America, 104(6), 3616–3625. 10.1121/1.423944 9857519

[ece38902-bib-0069] Stevenson, B. C. , Borchers, D. L. , Altwegg, R. , Swift, R. J. , Gillespie, D. M. , & Measey, G. J. (2015). A general framework for animal density estimation from acoustic detections across a fixed microphone array. Methods in Ecology and Evolution, 6, 38–48. 10.1111/2041-210X.12291

[ece38902-bib-0070] Tavolga, W. (2012). Listening backward: Early days of marine bioacoustics. In A. N. Popper & A. D. Hawkins (Eds.), The effects of noise on aquatic life (pp. 695). Springler‐Verlag. 10.1007/978-1-4419-7311-5 22278439

[ece38902-bib-0071] Theis, K. R. , Greene, K. M. , Benson‐Amram, S. R. , & Holekamp, K. E. (2007). Sources of variation in the long‐distance vocalizations of spotted hyenas. Behaviour, 144, 557–584. 10.1163/156853907780713046

[ece38902-bib-0072] Trikootam, S. C. , & Hornikx, M. (2019). The wind effect on sound propagation over urban areas: Experimental approach with an uncontrolled sound source. Building and Environment, 149, 561–570. 10.1016/j.buildenv.2018.11.037

[ece38902-bib-0073] Uhlenbroek, C. (1996). The structure and function of the long distance calls given by male chimpanzees in Gombe National Park. PhD Thesis. University of Bristol.

[ece38902-bib-0074] Urazghildiiev, I. R. , & Clark, C. W. (2013). Comparative analysis of localization algorithms with application to passive acoustic monitoring. The Journal of the Acoustical Society of America, 134(6), 4418–4426. 10.1121/1.4824683 25669253

[ece38902-bib-0075] Wang, H. , Chen, C. E. , Ali, A. , Asgari, S. , Hudson, R. E. , Yao, K. , & Taylor, C. (2005). Acoustic sensor networks for woodpecker localization. Advanced Signal Processing Algorithms, Architectures, and Implementations XV, 5910. 10.1117/12.617983

[ece38902-bib-0076] Wiggins, S. M. , Frasier, K. E. , Henderson, E. E. , & Hildebrand, J. A. (2013). Tracking dolphin whistles using an autonomous acoustic recorder array. The Journal of the Acoustical Society of America, 133(6), 3813–3818. 10.1121/1.4802645 23742335

[ece38902-bib-0077] Wijers, M. , Loveridge, A. , Macdonald, D. W. , & Markham, A. (2019). CARACAL: A versatile passive acoustic monitoring tool for wildlife research and conservation. Bioacoustics, 1–17, 10.1080/09524622.2019.1685408

[ece38902-bib-0078] Wildlife Acoustics. (2022). Wildlife acoustics online store webpage. Retrieved from https://www.wildlifeacoustics.com/products

[ece38902-bib-0079] Williamson, E. A. , & Feistner, A. T. C. (2003). Habituating primates: Processes, techniques, variables and ethics. In J. M. Setchell & D. J. Curis (Eds.), Field and laboratory methods in primatology: A practical guide (pp. 33–50). Cambridge University Press.

[ece38902-bib-0080] Wilson, D. R. , Battiston, M. , Brzustowski, J. , & Mennill, D. J. (2014). Sound Finder: A new software approach for localizing animals recorded with a microphone array. Bioacoustics, 23(2), 99–112. 10.1080/09524622.2013.827588

[ece38902-bib-0081] Wilson, M. L. , Hauser, M. D. , & Wrangham, R. W. (2007). Chimpanzees (*Pan troglodytes*) modify grouping and vocal behaviour in response to location‐specific risk. Behaviour, 144(12), 1621–1653. 10.1163/156853907782512137

[ece38902-bib-0082] Wilson, R. P. , & McMahon, C. R. (2006). Measuring devices on wild animals: What constitutes acceptable devices? Frontiers in Ecology and the Environment, 4(3), 147–154.

[ece38902-bib-0083] Wölfel, M. , & McDonough, J. (2009). Distant speech recognition. John Wiley & Sons.

[ece38902-bib-0084] Wrangham, R. W. (1974). Artificial feeding of chimpanzees and baboons in their natural habitat. Animal Behaviour, 22(1), 83–93. 10.1016/S0003-3472(74)80056-4

[ece38902-bib-0085] Wrege, P. H. , Rowland, E. D. , Keen, S. , & Shiu, Y. (2017). Acoustic monitoring for conservation in tropical forests: Examples from forest elephants. Methods in Ecology and Evolution, 8(10), 1292–1301. 10.1111/2041-210X.12730

